# Supplementary material for: Forecasting intermittent and sparse time series: A unified probabilistic framework via deep renewal processes
Source: PLoS One. 2021 Nov 29;16(11):e0259764. doi: 10.1371/journal.pone.0259764 (PMC8629246; doi:10.1371/journal.pone.0259764)
Supplement: S1 Appendix — (ZIP) [file pone.0259764.s001.zip › turkmenetal_r3_supplement_source.pdf]

A Supporting information

A.1 Negative Binomial Distribution.

We use a slightly altered version of the negative binomial distribution. Recall that the (generalized) negative binomial random variable is defined with the probability mass function

$$\mathbb{P}\{X = k\} = \binom{k+r-1}{k}(1-\pi)^r \pi^k,$$

where $k \in \{0, 1, 2, \dots\}, r > 0, \pi \in [0, 1]$. We "shift" the distribution for consistency with definitions of interarrival times and demand sizes. That is, we define $Y \in \{1, 2, \cdots\}$ such that $Y = X + 1$. We then have,

$$\mathbb{P}\{Y = k\} = \binom{k+r-2}{k-1}(1-\pi)^r \pi^{(k-1)}.$$

We also parameterize the distribution with the "mean-dispersion" convention, defining

$$\mu = \frac{\pi r}{1-\pi} + 1 \qquad \nu = \frac{1}{1-\pi},$$

with $\mu > 1, \nu > 1$. Note $\mathbb{E}[Y] = \mu$ and that $\mathbb{V}[Y]/(\mathbb{E}[Y] - 1) = \mathbb{V}[X]/\mathbb{E}[X] = \nu$ is the "shifted" variance-to-mean ratio. Concretely, when we define $Y \sim \mathcal{NB}(\mu, \nu)$, we refer to the random variable determined by the probability mass function

$$p(k) = \mathbb{P}\{Y = k\} = \binom{k + \frac{\mu-1}{\nu-1} - 2}{k-1}\left(\frac{1}{\nu}\right)^{\frac{\mu-1}{\nu-1}}\left(1 - \frac{1}{\nu}\right)^{(k-1)}.$$

The distribution function of Y is

$$F_Y(k) = \mathbb{P}\{Y \le k\} = 1 - I_{1-1/\nu}\left(k, \frac{\mu-1}{\nu-1}\right),$$

where $I_x(a, b)$ is the regularized *incomplete Beta function.*

Finally, let us give an explicit form for the *hazard rate* implied by negative binomial random variables,

$$h(k) = \frac{p(k)}{1-F(k-1)} = \frac{F(k)-F(k-1)}{1-F(k-1)} = \frac{I_{1-1/\nu}(k-1,r) - I_{1-1/\nu}(k,r)}{I_{1-1/\nu}(k-1,r)} \tag{1}$$

$$= 1 - \frac{I_{1-1/\nu}(k,r)}{I_{1-1/\nu}(k-1,r)}, \tag{2}$$

where we keep $r = \dfrac{\mu-1}{\nu-1}$ and take $F(0) = 0$.

A.2 Forecast Functions for Static NB Models.

Many of the models we use in this work do not admit closed forms for forecast functions, *i.e.*, efficient closed-form estimators for the conditional mean forecast. Notable exceptions are Static models with with negative binomial interdemand times. Indeed, an analytical expression for $\mathbb{E}[Y_{n+1}|\mathcal{H}_n]$ is possible, where use $\mathcal{H}_n$ to denote the filtration up to demand review period n. Let $Z_n = [\![Y_n > 0]\!]$ is a binary random variable that is 1 when period n has positive demand. We denote the number of demand points in $\mathcal{H}_n$ as i, *i.e.*, $i = \sum_{\nu=0}^{n} Z_\nu$. Then,

$$\mathbb{E}[Y_{n+1}|\mathcal{H}_n] = \mathbb{E}[Z_{n+1}M_{i+1}|\mathcal{H}_n] = \mathbb{E}[Z_{n+1}|\mathcal{H}_n]\,\mathbb{E}[M_{i+1}|\mathcal{H}_n] = \mathbb{E}[Z_{n+1}|\mathcal{H}_n]\,\mathbb{E}[M_{i+1}].$$

Here, the first equality is by definition. The second follows from our model assumption that demand sizes and intervals are independent. Finally, the third equality follows from our assumption that demand sizes are independent of the past. Moreover,

$$\mathbb{E}\left[Z_{n+1}|\mathcal{H}_n\right] = \mathbb{P}\{Z_{n+1} = 1|\mathcal{H}_n\} = \mathbb{P}\{Z_{n+1} = 1|T_i\},$$

where we define $T_i = \sum_{j=0}^{i} Q_j$ as the *time* of the previous nonzero demand. This follows from our renewal process assumption. That is, given the time of the previous issue point, the time of the next point is conditionally independent of the history. We can rewrite

$$\mathbb{P}\{Z_{n+1} = 1|T_i\} = \mathbb{P}\{Q_{i+1} = n - T_i + 1|Q_{i+1} > n - T_i\} = h_Q(n - T_i)$$

where h_Q is the *hazard rate* (1). Finally, we have

$$\mathbb{E}\left[Y_{n+1}|\mathcal{H}_n\right] = h_Q(n - T_i)\mathbb{E}\left[M_{i+1}\right].$$

Conditioning on the EWMA process, similar expressions can be derived easily for EWMA-type self-modulating DTRPs. Finally, tools from renewal theory can be used to characterize multi-step forecasts, see *e.g.*, discussions in [1].

A.3 Convergence Problem.

In the Models section, we introduced self-modulated DTRP models, and commented that these models suffered from a similar "convergence" issue as in previous *nonnegative EWMA* models [2, 3].

In our construction, we defined the size-interval sequence $\{(M_i, Q_i)\}$ on positive integers $\{1, 2, \cdots\}$. Therefore, in contrast to the "convergence to zero" issue outlined in, *e.g.*, [3], our models are plagued by convergence to dense trajectories of demand sizes 1. This can be a slightly more "desirable" problem, although we should still caution that these models are not suited for forecasts with long lead times.

More formally, let

$$M_i - 1 \sim \mathcal{PO}(\hat{M}_{i-1} - 1), \tag{3a}$$

$$\hat{M}_i = (1 - \beta)\hat{M}_{i-1} + \beta M_i, \tag{3b}$$

where $0 < \beta \leq 1$. Below, we give a statement of the "convergence to one" issue with an argument that follows [2], albeit with a slightly more accessible proof.

Proposition 1. *Let M_i be defined as in (3). $M_i \longrightarrow 1$ as $i \uparrow \infty$ almost surely.*

Proof. First note that $\{\hat{M}_i\}$ is a positive martingale, since $\mathbb{E}[\hat{M}_i|M_{1:i-1}] = \mathbb{E}[\hat{M}_i|\hat{M}_{i-1}] = \hat{M}_{i-1}$. By the martingale convergence theorem [4, Sec 7.4], $\hat{M}_i \to X$ a.s. for some random variable X, and $X \in [1, \infty)$ naturally. By (3b), it is also clear that $M_i \to X$ a.s.

However, we must then note $M_i - \hat{M}_{i-1} = M_i - \mathbb{E}[M_i] \to 0$. In other words, X is a degenerate random variable, taking a value in $[1, \infty)$ with probability 1. However, M_i is degenerate iff $\mathbb{V}[M_i] = 0$. Noting that M_i is defined as a shifted Poisson random variable, and $\mathbb{V}[M_i] \to \mathbb{V}[X] = X - 1 = 0$, we have the desired proof. □

Similarly, this proof can be extended to geometric and negative binomial random variables to show that

Corollary 1. *$Q_i \longrightarrow 1$ as $i \uparrow \infty$ a.s.*

Static models do not suffer from the convergence problem. Moreover, [5] discuss a set of models with stationary mean processes that mitigate this issue. This is done by defining both sizes and intervals as a stationary autoregressive process. For example,

$$\hat{M}_i = (1 - \varphi - \beta)\mu + \beta\hat{M}_{i-1} + \varphi M_i,$$

where $\varphi + \beta < 1$, $\varphi, \beta, \mu \in \mathbb{R}_+$, and Q_i analogously.

A.4 Forecast accuracy metrics.

Our first two forecast accuracy metrics are based on forecast distributions, while the rest are some of the most commonly used metrics for measuring point forecast accuracy.

- **P50** and **P90 Loss** are metrics based on the quantile loss. Letting $\hat{y}(\rho)$ denote the quantile estimate—here obtained via sampling from the forecast distribution—we denote

  $$\mathrm{QL}_\rho(y_n, \widehat{y}_n(\rho)) = \begin{cases} 2 \cdot \rho \cdot (y_n - \widehat{y}_n(\rho)), & y_n - \widehat{y}_n(\rho) > 0, \\ 2 \cdot (1-\rho) \cdot (\widehat{y}_n(\rho) - y_n), & y_n - \widehat{y}_n(\rho) \leqslant 0. \end{cases}$$

  We define **PXXLoss**, given lead time $L < N$,

  $$\mathrm{PXXLoss}_\rho(\mathbf{y}, \hat{\mathbf{y}}) = \frac{1}{LM} \sum_{i=1}^{M} \sum_{n=1}^{L} \mathrm{QL}_\rho\left(y_{in}, \hat{y}_{in}\right)$$

  We report **P50Loss** and **P90Loss**, setting $\rho = 0.5$ and $\rho = 0.9$ respectively.

- **RMSE** is root mean squared error.

  $$\mathrm{RMSE}(\mathbf{y}, \hat{\mathbf{y}}) = \left(\frac{1}{LM} \sum_{i=1}^{M} \sum_{n=1}^{L} (y_{in} - \hat{y}_{in})^2 \right)^{\frac{1}{2}}.$$

- Finally, **RMSSE** is root mean squared scaled error, also used in the M5 forecasting competition [6].

  $$\mathrm{RMSSE}(\mathbf{y}, \hat{\mathbf{y}}) = \frac{1}{ML} \sum_{i=1}^{M} \sum_{n=1}^{L} \frac{(y_{in} - \hat{y}_{in})^2}{\frac{1}{L'} \sum_{n'=1}^{L'-1} |y_{i,n'+1} - y_{in'}|}.$$

  Here, we let n' index the in-sample time series (before the forecast horizon), that has length L'.

References

1. Feller W. An introduction to probability theory and its applications. John Wiley & Sons; 1957.
2. Grunwald GK, Hamza K, Hyndman RJ. Some properties and generalizations of non-negative Bayesian time series models. Journal of the Royal Statistical Society: Series B (Statistical Methodology). 1997;59(3):615–626.
3. Shenstone L, Hyndman RJ. Stochastic models underlying Croston's method for intermittent demand forecasting. Journal of Forecasting. 2005;24(6):389–402.
4. Shiryaev AN. Probability. Springer; 1996.
5. Snyder RD, Ord JK, Beaumont A. Forecasting the intermittent demand for slow-moving inventories: A modelling approach. International Journal of Forecasting. 2012;28(2):485–496.
6. Makridakis S. The M5 Competition Competitors' Guide. University of Nicosia; 2020. Available from: `https://www.kaggle.com/c/m5-forecasting-accuracy/data`.
